# Boundary layer flow of nanofluid over an exponentially stretching surface

**DOI:** 10.1186/1556-276X-7-94

**Published:** 2012-01-30

**Authors:** Sohail Nadeem, Changhoon Lee

**Affiliations:** 1 Department of Mathematics, Quaid-i-Azam University, 45320, Islamabad 44000, Pakistan; 2 Department of Computational Science and Engineering, Yonsei University, Seoul, Korea

**Keywords:** nanofluid, porous stretching surface, boundary layer flow, series solutions, exponential stretching

## Abstract

The steady boundary layer flow of nanofluid over an exponential stretching surface is investigated analytically. The transport equations include the effects of Brownian motion parameter and thermophoresis parameter. The highly nonlinear coupled partial differential equations are simplified with the help of suitable similarity transformations. The reduced equations are then solved analytically with the help of homotopy analysis method (HAM). The convergence of HAM solutions are obtained by plotting *h*-curve. The expressions for velocity, temperature and nanoparticle volume fraction are computed for some values of the parameters namely, suction injection parameter *α*, Lewis number *Le*, the Brownian motion parameter *Nb *and thermophoresis parameter *Nt*.

## 1 Introduction

During the last many years, the study of boundary layer flow and heat transfer over a stretching surface has achieved a lot of success because of its large number of applications in industry and technology. Few of these applications are materials manufactured by polymer extrusion, drawing of copper wires, continuous stretching of plastic films, artificial fibers, hot rolling, wire drawing, glass fiber, metal extrusion and metal spinning etc. After the pioneering work by Sakiadis [1], a large amount of literature is available on boundary layer flow of Newtonian and non-Newtonian fluids over linear and nonlinear stretching surfaces [2-10]. However, only a limited attention has been paid to the study of exponential stretching surface. Mention may be made to the works of Magyari and Keller [11], Sanjayanand and Khan [12], Khan and Sanjayanand [13], Bidin and Nazar [14] and Nadeem et al. [15,16].

More recently, the study of convective heat transfer in nanofluids has achieved great success in various industrial processes. A large number of experimental and theoretical studies have been carried out by numerous researchers on thermal conductivity of nanofluids [17-22]. The theory of nanofluids has presented several fundamental properties with the large enhancement in thermal conductivity as compared to the base fluid [23].

In this study, we have discussed the boundary layer flow of nanofluid over an exponentially stretching surface with suction and injection. To the best of our knowledge, the nanofluid over an exponentially stretching surface has not been discussed so far. However, the present paper is only a theoretical idea, which is not checked experimentally. The governing highly nonlinear partial differential equation of motion, energy and nanoparticle volume fraction has been simplified by using suitable similarity transformations and then solved analytically with the help of HAM [24-39]. The convergence of HAM solution has been discussed by plotting *h*-curve. The effects of pertinent parameters of nanofluid have been discussed through graphs.

## 2 Formulation of the problem

Consider the steady two-dimensional flow of an incompressible nanofluid over an exponentially stretching surface. We are considering Cartesian coordinate system in such a way that *x*-axis is taken along the stretching surface in the direction of the motion and y-axis is normal to it. The plate is stretched in the *x*-direction with a velocity *U_w _*= *U*_0 _exp (*x*/*l*). defined at *y *= 0. The flow and heat transfer characteristics under the boundary layer approximations are governed by the following equations

(1) $$\frac{\partial u}{\partial x}+\frac{\partial v}{\partial y}=0,$$

(2) $$u\frac{\partial u}{\partial x}+v\frac{\partial u}{\partial y}=\nu \frac{\partial^{2}u}{\partial y^{2}},$$

(3) $$u\frac{\partial T}{\partial x}+v\frac{\partial T}{\partial y}=\alpha \frac{\partial^{2}T}{\partial y^{2}}+\frac{{\left(\rho c\right)}_{p}}{{\left(\rho c\right)}_{f}}\left[D_{B}\frac{\partial C}{\partial y}\frac{\partial T}{\partial y}+\frac{D_{T}}{T_{\infty }}{\left(\frac{\partial T}{\partial y}\right)}^{2}\right],$$

(4) $$u\frac{\partial C}{\partial x}+v\frac{\partial C}{\partial y}=D_{B}\frac{\partial^{2}C}{\partial y^{2}}+\frac{D_{T}}{T_{\infty }}\left(\frac{\partial^{2}T}{\partial y^{2}}\right),$$

where (*u*, *v*) are the velocity components in (*x*, *y*) directions, *ρ_f _*is the fluid density of base fluid, *ν *is the kinematic viscosity, *T *is the temperature, *C *is the nanoparticle volume fraction, (*ρc*)*_p _*is the effective heat capacity of nanoparticles, (*ρc*)*_f _*is the heat capacity of the fluid, *α *= *k*/(*ρc*)*_f _*is the thermal diffusivity of the fluid, *D_B _*is the Brownian diffusion coefficient and *D_T _*is the thermophoretic diffusion coefficient.

The corresponding boundary conditions for the flow problem are

(5) $$\begin{array}{c} u=U_{w}\left(x\right)=U_{0}exp\left(x/l\right),\;v=-\beta \left(x\right),\;T=T_{w},\;C=C_{w}\;at\;y=0,\\ u=0,\;T=T_{\infty }\;C=C_{\infty }\;as\;y\to \infty , \end{array}$$

in which *U*_0 _is the reference velocity, *β*(*x*) is the suction and injection velocity when *β*(*x*) > 0 and *β*(*x*) < 0, respectively, *T_w _*and *T*_∞ _are the temperatures of the sheet and the ambient fluid, *C_w_*, *C*_∞ _are the nanoparticles volume fraction of the plate and the fluid, respectively.

We are interested in similarity solution of the above boundary value problem; therefore, we introduce the following similarity transformations

(6) $$\begin{array}{c} u=U_{0}exp\left(\frac{x}{l}\right)f^{\prime }\left(\eta \right),\;v=-\sqrt{\frac{vU_{0}}{2l}}exp\left(\frac{x}{2l}\right)\left\{f\left(\eta \right)+\eta f^{\prime }\left(\eta \right)\right\},\\ \eta =y\sqrt{\frac{U_{0}}{2vl}}exp\left(\frac{x}{2l}\right),\;\theta =\frac{T-T_{\infty }}{T_{w}-T_{\infty }},\;g=\frac{C-C_{\infty }}{C_{w}-C_{\infty }}. \end{array}$$

Making use of transformations (6), Eq. (1) is identically satisfied and Equations (2)-(4) take the form

(7) $$f_{\eta \eta \eta }+ff_{\eta \eta }-2f_{\eta }^{2}=0,$$

(8) $$\theta_{\eta \eta }+Pr\left(f\theta_{\eta }-f_{\eta }\theta +Nb\theta_{\eta }g_{\eta }+Nt\theta_{\eta }^{2}\right)=0$$

(9) $$g_{\eta \eta }+Le\left(fg_{\eta }-f_{\eta }g\right)+\frac{Nt}{Nb}\theta_{\eta \eta }=0,$$

(10) $$\begin{array}{c} f=-v_{w},\;f_{\eta }=1,\;\theta =1,\;g=1\;at\;\eta =0,\\ f_{\eta }\to 0,\;\theta \to 0,\;g\to 0\;as\;\eta \to \infty , \end{array}$$

where

$$Nt=D_{B}\frac{{\left(\rho c\right)}_{p}}{{\left(\rho c\right)}_{f}}\left(C_{w}-C_{\infty }\right),\;\;Nb=\frac{D_{T}}{T_{\infty }}\frac{{\left(\rho c\right)}_{p}}{{\left(\rho c\right)}_{f}}\frac{\left(T_{w}-T_{\infty }\right)}{\upsilon },\;Le=\frac{\upsilon }{D_{B}},\;Pr=\frac{\upsilon }{\alpha }.$$

The physical quantities of interest in this problem are the local skin-friction coefficient *C_f_*, Nusselt number *Nu_x _*and the local Sherwood number *Sh_x_*, which are defined as(11)

where Re*_x _*= *U_w_x*/*ν *is the local Renolds number.

## 3 Solution by homotopy analysis method

For HAM solutions, the initial guesses and the linear operators *L_i _*(*i *= 1 - 3) are

(12) $$f_{0}\left(\eta \right)=1-v_{w}-{\text{e}}^{-\eta },\;\theta_{0}\left(\eta \right)={\text{e}}^{-\eta },\;g_{0}\left(\eta \right)={\text{e}}^{-\eta },$$

(13) $${ℒ}_{1}\left(f\right)=f^{\prime \prime \prime }-f^{\prime },\;{ℒ}_{2}\left(\theta \right)=\theta^{\prime \prime }-\theta ,\;{ℒ}_{3}\left(g\right)=g^{\prime \prime }-g.$$

The operators satisfy the following properties

(14) $${ℒ}_{1}\left[c_{1}{\text{e}}^{-\eta }+c_{2}{\text{e}}^{\eta }+c_{3}\right]=0,$$

(15) $${ℒ}_{2}\left[c_{4}{\text{e}}^{-\eta }+c_{5}{\text{e}}^{\eta }\right]=0,$$

(16) $${ℒ}_{3}\left[c_{6}{\text{e}}^{-\eta }+c_{7}{\text{e}}^{\eta }\right]=0,$$

in which *C*_1 _to *C*_7 _are constants. From Equations (7) *to *(9), we can define the following zeroth-order deformation problems

(17) $$\left(1-p\right){ℒ}_{1}\left[\hat{f}\left(\eta ,p\right)-f_{0}\left(\eta \right)\right]=p\hbar_{1}H_{1}{Ñ}_{1}\left[\hat{f}\left(\eta ,p\right)\right],$$

(18) $$\left(1-p\right){ℒ}_{2}\left[\hat{\theta }\left(\eta ,p\right)-\theta_{0}\left(\eta \right)\right]=p\hbar_{2}H_{2}{Ñ}_{2}\left[\hat{\theta }\left(\eta ,p\right)\right],$$

(19) $$\left(1-p\right){ℒ}_{3}\left[ĝ\left(\eta ,p\right)-g_{0}\left(\eta \right)\right]=p\hbar_{3}H_{3}{Ñ}_{3}\left[ĝ\left(\eta ,p\right)\right],$$

(20) $$\hat{f}\left(0,p\right)=-v_{w},\;{\hat{f}}^{\prime }\left(0,p\right)=1,\;{\hat{f}}^{\prime }\left(\infty ,p\right)=0,$$

(21) $$\hat{\theta }\left(0,p\right)=1,\;\hat{\theta }\left(\infty ,p\right)=0,$$

(22) $${ĝ}^{\prime }\left(0,p\right)=1,\;ĝ\left(\infty ,p\right)=0.$$

In Equations (17)-(22), *ħ*_1_, *ħ*_2_, and *ħ*_3 _denote the non-zero auxiliary parameters, *H*_1_, *H*_2 _and *H*_3 _are the non-zero auxiliary function (*H*_1 _= *H*_2 _= *H*_3 _= 1) and

(23) $${Ñ}_{1}\left[\hat{f}\left(\eta ,p\right)\right]=\frac{\partial^{3}f}{\partial \eta^{3}}-2{\left(\frac{\partial f}{\partial \eta }\right)}^{2}+f\frac{\partial^{2}f}{\partial \eta^{2}},$$

(24) $${Ñ}_{2}\left[\hat{\theta }\left(\eta ,p\right)\right]=\frac{\partial^{2}\theta }{\partial \eta^{2}}+Pr\left(f\frac{\partial \theta }{\partial \eta }-\frac{\partial f}{\partial \eta }\theta +Nb\frac{\partial \theta }{\partial \eta }\frac{\partial g}{\partial \eta }+Nt{\left(\frac{\partial \theta }{\partial \eta }\right)}^{2}\right),$$

(25) $${Ñ}_{3}\left[ĝ\left(\eta ,p\right)\right]=\frac{\partial^{2}g}{\partial \eta^{2}}+Le\left(f\frac{\partial g}{\partial \eta }-\frac{\partial f}{\partial \eta }g+\frac{Nt}{Nb}\theta_{\eta \eta }\right)+\frac{Nt}{Nb}\frac{\partial^{2}\theta }{\partial \eta^{2}}.$$

Obviously

(26) $$\hat{f}\left(\eta ,0\right)=f_{0}\left(\eta \right),\;\hat{f}\left(\eta ,1\right)=f\left(\eta \right),$$

(27) $$\hat{\theta }\left(\eta ,0\right)=\theta_{0}\left(\eta \right),\;\hat{\theta }\left(\eta ,1\right)=\theta \left(\eta \right),$$

(28) $$ĝ\left(\eta ,0\right)=g_{0}\left(\eta \right),\;ĝ\left(\eta ,1\right)=g\left(\eta \right).$$

When *p *varies from 0 to 1, then $\hat{f}\left(\eta ,p\right)$, $\hat{\theta }\left(\eta ,p\right)$, $ĝ\left(\eta ,p\right)$ vary from initial guesses *f*_0 _(*η*), *θ*_0 _(*η*) and *g*_0 _(*η*) to the final solutions *f *(*η*), *θ *(*η*) and *g *(*η*), respectively. Considering that the auxiliary parameters *ħ*_1_, *ħ*_2 _and *ħ*_3 _are so properly chosen that the Taylor series of $\hat{f}\left(\eta ,p\right)$, $\hat{\theta }\left(\eta ,p\right)$ and $ĝ\left(\eta ,p\right)$ expanded with respect to an embedding parameter converge at *p *= 1, hence Equations (17)-(19) become

(29) $$\hat{f}\left(\eta ,p\right)=f_{0}\left(\eta \right)+\overset{\infty }{\underset{m=1}{\sum }}f_{m}\left(\eta \right)p^{m},$$

(30) $$\hat{\theta }\left(\eta ,p\right)=\theta_{0}\left(\eta \right)+\overset{\infty }{\underset{m=1}{\sum }}\theta_{m}\left(\eta \right)p^{m},$$

(31) $$ĝ\left(\eta ,p\right)=g_{0}\left(\eta \right)+\overset{\infty }{\underset{m=1}{\sum }}g_{m}\left(\eta \right)p^{m},$$

(32) $$f_{m}\left(\eta \right)=\frac{1}{m!}{\left(\frac{\partial^{m}\hat{f}\left(\eta ,p\right)}{\partial p^{m}}\right|}_{p=0},$$

(33) $$\theta_{m}\left(\eta \right)=\frac{1}{m!}{\left(\frac{\partial^{m}\hat{\theta }\left(\eta ,p\right)}{\partial p^{m}}\right|}_{p=0},$$

(34) $$g_{m}\left(\eta \right)=\frac{1}{m!}{\left(\frac{\partial^{m}ĝ\left(\eta ,p\right)}{\partial p^{m}}\right|}_{p=0}.$$

The mth-order problems are defined as follow

(35) $${ℒ}_{1}\left[f_{m}\left(\eta \right)-\chi_{m}f_{m-1}\left(\eta \right)\right]=\hbar_{1}{Ř}_{m}^{1}\left(\eta \right),$$

(36) $${ℒ}_{2}\left[\theta_{m}\left(\eta \right)-\chi_{m}\theta_{m-1}\left(\eta \right)\right]=\hbar_{2}{Ř}_{m}^{2}\left(\eta \right),$$

(37) $${ℒ}_{3}\left[g_{m}\left(\eta \right)-\chi_{m}g_{m-1}\left(\eta \right)\right]=\hbar_{3}{Ř}_{m}^{3}\left(\eta \right),$$

(38) $$f_{m}\left(0\right)=f_{m}^{\prime }\left(0\right)=f_{m}^{\prime }\left(\infty \right)=0,$$

(39) $$\theta_{m}\left(0\right)=\theta_{m}\left(\infty \right)=0,$$

(40) $$g_{m}^{\prime }\left(0\right)=g_{m}\left(\infty \right)=0,$$

where

(41) $$\chi_{m}=\left\{\begin{array}{c} 0,\;m\leq 1,\\ 1,\;m>1. \end{array}\right)$$

(42) $${Ř}_{m}^{1}\left(\eta \right)=f_{m-1}^{\prime \prime \prime }\left(\eta \right)+\overset{m-1}{\underset{k=0}{\sum }}f_{m-1-k}f_{k}^{\prime \prime }-2\overset{m-1}{\underset{k=0}{\sum }}f_{m-1-k}^{\prime }f_{k}^{\prime },$$

(43) $${Ř}_{m}^{2}\left(\eta \right)=\theta_{m-1}^{\prime \prime }+Pr\overset{m-1}{\underset{k=0}{\sum }}\left\{f_{m-1-k}\theta_{k}^{\prime }-f_{m-1-k}^{\prime }\theta_{k}+Nb\theta_{m-1-k}^{\prime }g_{k}^{\prime }+Nt\theta_{m-1-k}^{\prime }\theta_{k}^{\prime }\right\},$$

(44) $${Ř}_{m}^{3}\left(\eta \right)=g_{m-1}^{\prime \prime }+Le\overset{m-1}{\underset{k=0}{\sum }}\left\{f_{m-1-k}g_{k}^{\prime }-f_{m-1-k}^{\prime }g_{k}\right\}+\frac{Nt}{Nb}\theta_{m-1}^{\prime \prime }.$$

Employing MATHEMATICA, Equations (35)-(40) have the following solutions

(45) $$f\left(\eta \right)=\overset{\infty }{\underset{m=0}{\sum }}f_{m}\left(\eta \right)=\underset{M\to \infty }{\lim}\left[\overset{M}{\underset{m=0}{\sum }}\;a_{m,0}^{0}+\overset{M+1}{\underset{n=1}{\sum }}{\text{e}}^{-n\eta }\left(\overset{M}{\underset{m=n-1}{\sum }}\overset{m+1-n}{\underset{k=0}{\sum }}a_{m,n}^{k}\eta^{k}\right)\right],$$

(46) $$\theta \left(\eta \right)=\overset{\infty }{\underset{m=0}{\sum }}\theta_{m}\left(\eta \right)=\underset{M\to \infty }{\lim}\left[\overset{M+2}{\underset{n=1}{\sum }}{\text{e}}^{-n\eta }\left(\overset{M+1}{\underset{m=n-1}{\sum }}\overset{m+1-n}{\underset{k=0}{\sum }}A_{m,n}^{k}\eta^{k}\right)\right],$$

(47) $$g\left(\eta \right)=\overset{\infty }{\underset{m=0}{\sum }}g_{m}\left(\eta \right)=\underset{M\to \infty }{\lim}\left[\overset{M+2}{\underset{n=1}{\sum }}{\text{e}}^{-n\eta }\left(\overset{M+1}{\underset{m=n-1}{\sum }}\overset{m+1-n}{\underset{k=0}{\sum }}F_{m,n}^{k}\eta^{k}\right)\right],$$

in which $a_{m,0}^{0}$, $a_{m,n}^{k}$, $A_{m,n}^{k}$, $F_{m,n}^{k}$ are the constants and the numerical data of above solutions are shown through graphs in the following section.

## 4 Results and discussion

**Figure 1:**
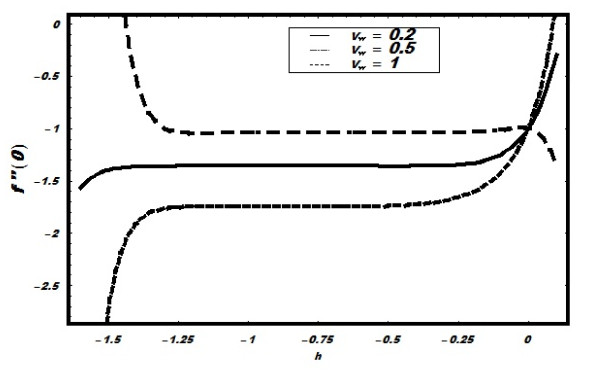
***h*-Curve for velocity**.

**Figure 2:**
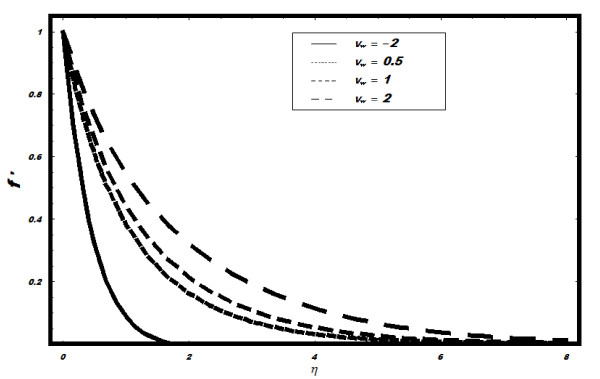
**Velocity for different values of suction and injection parameter**.

**Figure 3:**
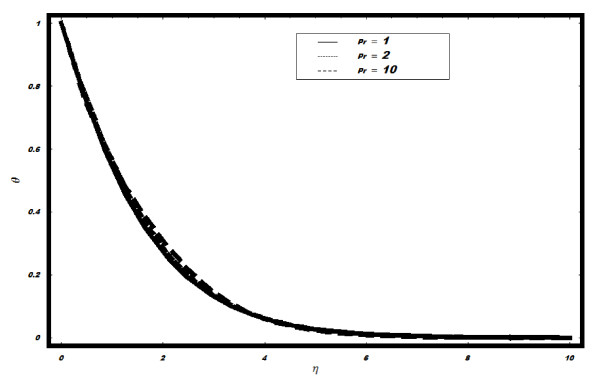
**Variation of temperature for different values of Pr when *Le *= 2, *h *= -0.1, *Nt *= *Nb *= 0.5, *v_w_*= 1**.

**Figure 4:**
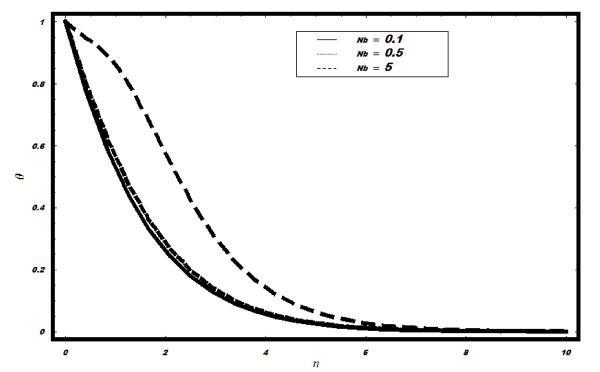
**Variation of temperature for different values of *Nb *when *Le *= 2, *h *= -0.1, *Nt *= 0.5, *v_w _*= 1, Pr = 2**.

**Figure 5:**
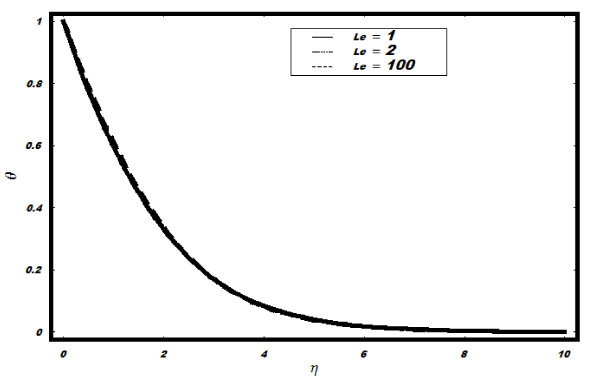
**Variation of temperature for different values of *Le *when *h *= -0.1, *Nt *= *Nb *= 0.5, *v_w _*= 1, Pr = 2**.

**Figure 6:**
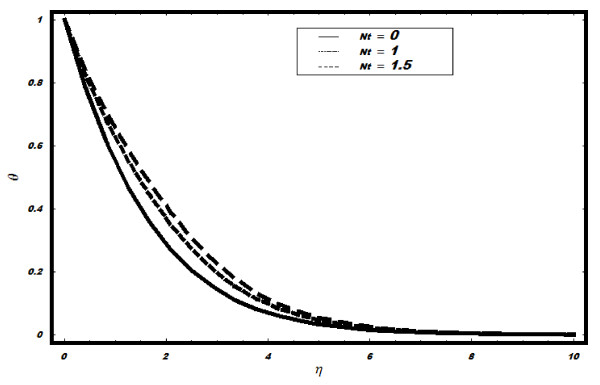
**Variation of temperature for different values of *Nt *when *Le *= 2, *h *= -0.1, *Nb *= 0.5, *v_w _*= 1, Pr = 2**.

**Figure 7:**
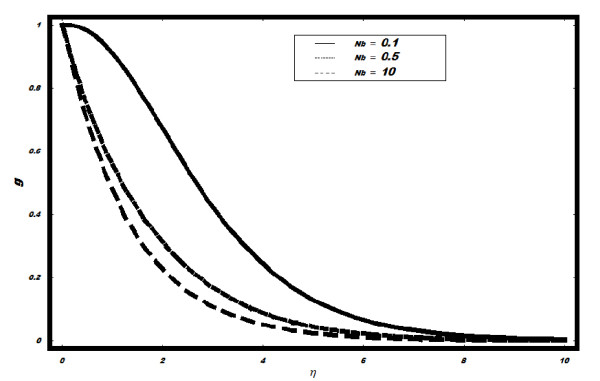
**Variation of nanoparticle fraction *g *for different values of *Nb *when *Le *= 2, *h *= -0.1, *Nt *= 0.5, *v_w _*= 1, Pr = 2**.

**Figure 8:**
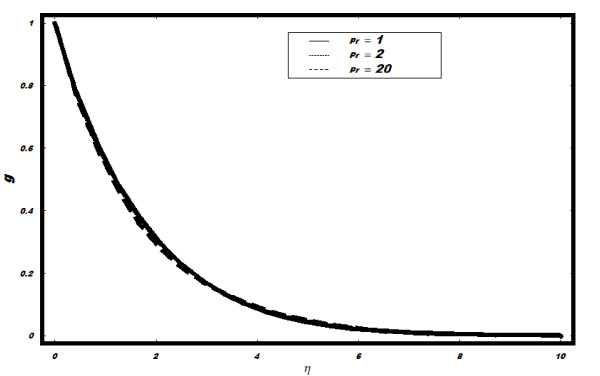
**Variation of nanoparticle fraction *g *for different values of Pr when *Le *= 2, *h *= -0.1, *Nt *= 0.5, *v_w _*= 1, *Nb *= 0.5**.

**Figure 9:**
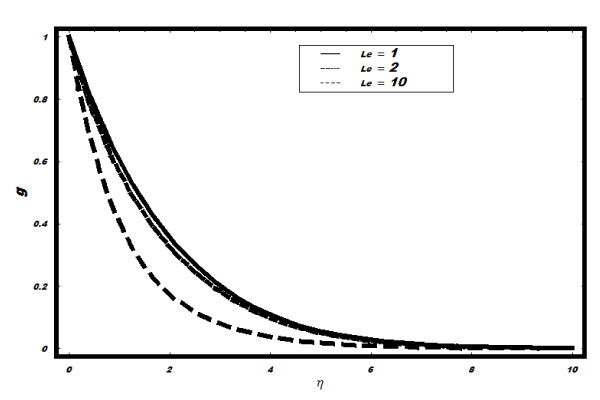
**Variation of nanoparticle fraction *g *for different values of *Le *when Pr = 2, *h *= -0.1, *Nt *= 0.5, *v_w _*= 1, *Nb *= 0.5**.

**Figure 10:**
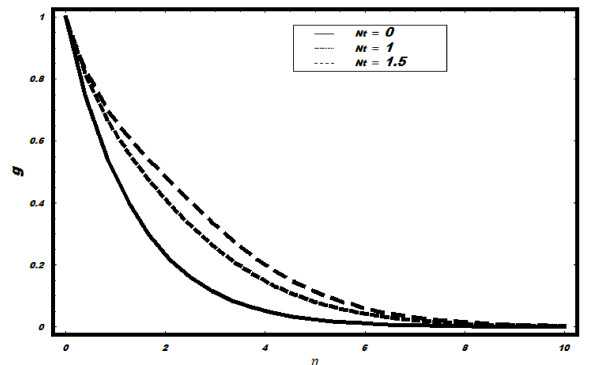
**Variation of nanoparticle fraction *g *for different values of *Nt *when *Le *= 2, *h *= -0.1, *Nt *= 0.5, *v_w _*= 1, Pr = 2**.

The numerical data of the solutions (45)-(47), which is obtained with the help of Mathematica, have been discussed through graphs. The convergence of the series solutions strongly depends on the values of non-zero auxiliary parameters *ħ_i _*(*i *= 1, 2, 3, *h*_1 _= *h*_2 _= *h*_3_), which can adjust and control the convergence of the solutions. Therefore, for the convergence of the solution, the *ħ*-curves is plotted for velocity field in Figure 1. We have found the convergence region of velocity for different values of suction injection parameter *v_w_*. It is seen that with the increase in suction parameter *v_w_*, the convergence region become smaller and smaller. Almost similar kind of convergence regions appear for temperature and nanoparticle volume fraction, which are not shown here. The non-dimensional velocity *f*′ against *η *for various values of suction injection parameter is shown in Figure 2. It is observed that velocity field increases with the increase in *v_w_*. Moreover, the suction causes the reduction of the boundary layer. The temperature field *θ *for different values of Prandtle number Pr, Brownian parameter *Nb*, Lewis number *Le *and thermophoresis parameter *Nt *is shown in Figures 3, 4, 5 and 6. In Figure 3, the temperature is plotted for different values of Pr. It is observed that with the increase in Pr, there is a very slight change in temperature; however, for very large Pr, the solutions seem to be unstable, which are not shown here. The variation of *Nb *on *θ *is shown in Figure 4. It is depicted that with the increase in *Nb*, the temperature profile increases. There is a minimal change in *θ *with the increase in *Le *(see Figure 5). The results remain unchanged for very large values of *Le*. The effects of *Nt *on *θ *are seen in Figure 6. It is seen that temperature profile increases with the increase in *Nt*; however, the thermal boundary layer thickness reduces. The nanoparticle volume fraction *g *for different values of Pr, *Nb*, *Nt *and *Le *is plotted in Figures 7, 8, 9 and 10. It is observed from Figure 7 that with the increase in *Nb*, g decreases and boundary layer for *g *also decreases. The effects of Pr on *g *are minimal. (See Figure 8). The effects of *Le *on *g *are shown in Figure 9. It is observed that *g *decreases as well as layer thickness reduces with the increase in *Le*. However, with the increase in *Nt*, *g *increases and layer thickness reduces (See Figure 10).

## Competing interests

This is just the theoretical study, every experimentalist can check it experimentally with our consent.

## Authors' contributions

SN done the major part of the article; however, the funding and computational suggestions and proof reading has been done by CL. All authors read and approved the final manuscript.

## References

[B1] Sakiadis BC Boundary layer behavior on continuous solid surfaces: I Boundary layer equations for two dimensional and axisymmetric flow AIChE J 1961 7 26 28

[B2] Liu IC Flow and heat transfer of an electrically conducting fluid of second grade over a stretching sheet subject to a transverse magnetic field Int J Heat Mass Transf 2004 7 4427 4437

[B3] Vajravelu K Rollins D Heat transfer in electrically conducting fluid over a stretching surface Int J Non-Linear Mech 1992 7 2 265 277

[B4] Vajravelu K Nayfeh J Convective heat transfer at a stretching sheet Acta Mech 1993 7 1-4 47 54

[B5] Khan SK Subhas Abel M Sonth Ravi M Viscoelastic MHD flow, heat and mass transfer over a porous stretching sheet with dissipation of energy and stress work Int J Heat Mass Transf 2003 7 47 57

[B6] Cortell R Effects of viscous dissipation and work done by deformation on the MHD flow and heat transfer of a viscoelastic fluid over a stretching sheet Phys Lett A 2006 7 298 305

[B7] Dandapat BS Santra B Vajravelu K The effects of variable fluid properties and thermocapillarity on the flow of a thin film on an unsteady stretching sheet Int J Heat Mass Transf 2007 7 991 996

[B8] Nadeem S Hussain A Malik MY Hayat T Series solutions for the stagnation flow of a second-grade fluid over a shrinking sheet Appl Math Mech Engl Ed 2009 7 1255 1262

[B9] Nadeem S Hussain A Khan M HAM solutions for boundary layer flow in the region of the stagnation point towards a stretching sheet Comm Nonlinear Sci Numer Simul 2010 7 475 481

[B10] Afzal N Heat transfer from a stretching surface Int J Heat Mass Transf 1993 7 1128 1131

[B11] Magyari E Keller B Heat and mass transfer in the boundary layer on an exponentially stretching continuous surface J Phys D Appl Phys 1999 7 577 785

[B12] Sanjayanand E Khan SK On heat and mass transfer in a viscoelastic boundary layer flow over an exponentially stretching sheet Int J Therm Sci 2006 7 819 828

[B13] Khan SK Sanjayanand E Viscoelastic boundary layer flow and heat transfer over an exponential stretching sheet Int J Heat Mass Transf 2005 7 1534 1542

[B14] Bidin B Nazar R Numerical solution of the boundary layer flow over an exponentially stretching sheet with thermal radiation Eur J Sci Res 2009 7 710 717

[B15] Nadeem S Hayat T Malik MY Rajput SA Thermal radiations effects on the flow by an exponentially stretching surface: a series solution Zeitschrift fur Naturforschung 2010 7 1 9

[B16] Nadeem S Zaheer S Fang T Effects of thermal radiations on the boundary layer flow of a Jeffrey fluid over an exponentially stretching surface Numer Algor 2011 7 187 205

[B17] Bachok N Ishak A Pop I boundary Layer flow of nanofluid over a moving surface in a flowing fluid Int J Therm Sci 2010 7 1663 1668

[B18] Choi SUS Siginer DA, Wang HP Enhancing thermal conductivity of fluids with nanoparticle Developments and Applications of Non-Newtonian Flows 1995 7 99 105 ASME FED 231/MD

[B19] Khanafer K Vafai K Lightstone M Buoyancy driven heat transfer enhancement in a two dimensional enclosure utilizing nanofluids Int J Heat Mass Transf 2003 7 3639 3653

[B20] Makinde OD Aziz A Boundary layer flow of a nano fluid past a stretching sheet with a convective boundary condition Int J Therm Sci 2011 7 1326 1332

[B21] Bayat J Nikseresht AH Investigation of the different base fluid effects on the nanofluids heat transfer and pressure drop Heat Mass Transfdoi:10.1007/s00231-011-0773-0

[B22] Hojjat M Etemad SG Bagheri R Laminar heat transfer of nanofluid in a circular tube Korean J Chem Eng 2010 7 5 1391 *1396

[B23] Fan J Wang L Heat conduction in nanofluids: structure-property correlation Int J Heat Mass Transf 2011 7 4349 4359

[B24] Liao SJ Beyond Perturbation Introduction to Homotopy Analysis Method 2003 Boca Raton: Chapman & Hall/CRC Press

[B25] Abbasbandy S The application of homotopy analysis method to nonlinear equations arising in heat transfer Phys Lett A 2006 7 109 113

[B26] Abbasbandy S Homotopy analysis method for heat radiation equations Int Commun Heat Mass Transf 2007 7 380 387

[B27] Abbasbandy S Tan Y Liao SJ Newton-homotopy analysis method for nonlinear equations Appl Math Comput 2007 7 1794 1800

[B28] Abbasbandy S Approximate solution for the nonlinear model of diffusion and reaction in porous catalysts by means of the homotopy analysis method Chem Eng J 2008 7 144 150

[B29] Abbasbandy S Soliton solutions for the Fitzhugh-Nagumo equation with the homotopy analysis method Appl Math Model 2008 7 2706 2714

[B30] Tan Y Abbasbandy S Homotopy analysis method for quadratic Ricati differential equation Comm Non-linear Sci Numer Simm 2008 7 539 546

[B31] Alomari AK Noorani MSM Nazar R Adaptation of homotopy analysis method for the numeric-analytic solution of Chen system Commun Nonlinear Sci Numer Simulat 2008 doi:10.1016/j.cnsns.2008.06.011

[B32] Rashidi MM Domairry G Dinarvand S Approximate solutions for the Burger and regularized long wave equations by means of the homotopy analysis method Commun Nonlinear Sci Numer Simul 2009 7 708 717

[B33] Chowdhury MSH Hashim I Abdulaziz O Comparison of homotopy analysis method and homotopy-perturbation method for purely nonlinear fin-type problems Commun Nonlinear Sci Numer Simul 2009 7 371 378

[B34] Sami Bataineh A Noorani MSM Hashim I On a new reliable modification of homotopy analysis method Commun Nonlinear Sci Numer Simul 2009 7 409 423

[B35] Sami Bataineh A Noorani MSM Hashim I Modified homotopy analysis method for solving systems of second-order BVPs Commun Nonlinear Sci Numer Simul 2009 7 430 442

[B36] Sami Bataineh A Noorani MSM Hashim I Solving systems of ODEs by homotopy analysis method Commun Nonlinear Sci Numer Simul 2008 7 2060 2070

[B37] Sajid M Ahmad I Hayat T Ayub M Series solution for unsteady axisymmetric flow and heat transfer over a radially stretching sheet Commun Nonlinear Sci Numer Simul 2008 7 2193 2202

[B38] Nadeem S Hussain A MHD flow of a viscous fluid on a non linear porous shrinking sheet with HAM Appl Math Mech Engl Ed 2009 7 1 10

[B39] Nadeem S Abbasbandy S Hussain M Series solutions of boundary layer flow of a Micropolar fluid near the stagnation point towards a shrinking sheet Z Naturforch 2009 7 575 582

